# Enhanced soil function and health by soybean root microbial communities during *in situ* remediation of Cd-contaminated soil with the application of soil amendments

**DOI:** 10.1128/msystems.01049-22

**Published:** 2023-05-18

**Authors:** Zhongyi Cheng, Jiachun Shi, Yan He, Yuxuan Chen, Youjing Wang, Xueling Yang, Tianyu Wang, Laosheng Wu, Jianming Xu

**Affiliations:** 1 Institute of Soil and Water Resources and Environmental Science, College of Environmental and Resource Sciences, Zhejiang University, Hangzhou, China; 2 Zhejiang Provincial Key Laboratory of Agricultural Resources and Environment, Hangzhou, China; 3 Department of Environmental Sciences, University of California, Riverside, California, USA; Institute of Soil Science Chinese Academy of Sciences, Nanjing, China

**Keywords:** cadmium-contaminated soil, soil amendment, soybean, multitrophic community assembly, multifunctionality, disease suppression

## Abstract

The interactions between soil microbiomes at various trophic levels are essential for restoring soil functions. Legumes are considered as “pioneer crops” in degraded or contaminated soils because they can fix nitrogen through symbiotic relationships with rhizobacteria, which promotes soil fertility. However, little is known about the abilities of legumes to contribute to the health of soil contaminated with cadmium (Cd). In this research, we applied a soil amendment (commercial Mg–Ca–Si conditioner, CMC) at two rates (1,500 and 3,000 kg/ha) in a Cd-contaminated soybean field. Bulk and rhizosphere soil samples were collected to assess the amendment-induced effects on four microbial lineages (bacteria, fungi, arbuscular mycorrhizal fungi [AMF], and nematodes) and their functions including Cd stabilization, nutrient cycling, and pathogen control. Compared with the control, both CMC application rates increased the pH and reduced labile Cd fraction in the bulk and rhizosphere soils. Although the total Cd concentrations in the soil were similar, the Cd accumulation in the grains was significantly reduced in treatments of soil amendments. It was observed that the application of CMC can significantly reduce the AMF diversity but increased the diversity of the other three communities. Moreover, the biodiversity within keystone modules (identified by co-occurrence network analysis) played key roles in driving soil multifunctionality. Specifically, key beneficial groups in module 2 such as *Aggregicoccus* (bacteria), Sordariomycetes (fungi), *Glomus* (AMF), and *Bursaphelenchus* (nematode) were strongly associated with soil multifunctionality. By co-culturing bacterial suspensions with the soybean root rot pathogen *Fusarium solani* in the *in vitro* assays, we experimentally validated that the application of CMC promoted the suppression of soil bacterial community on pathogens by inhibiting the mycelium growth and spore germination. Also, the bacterial community was more resistant to Cd stress in soils receiving CMC amendment. Our findings provide valuable theoretical references for enhancing soil functions and health via applying a soil amendment (CMC) during Cd-contaminated soil remediation.

**IMPORTANCE:**

Restoration of microbiome-driven soil functions and health is of great importance during Cd-contaminated soil remediation via soil amendment. Soybean and its symbiotic mutualism can provide abundant nitrogen and phosphorus to relieve the nutrient deficiency of Cd-contaminated soil. This study provides a novel perspective on the potential role of applying a soil amendment (CMC) in enhancing the functions and health of Cd-contaminated soils. Our results showed the distinct differences in soil microbial community responding to amendment-induced changes in edaphic properties. The biodiversity within keystone modules had major contributions to the maintenance of the soil’s multifunctionality and health. Additionally, a higher CMC application rate showed more beneficial effects. Collectively, our results enhance our understanding about the effects of applying CMC, together with soybean rotation, to enhance and maintain soil functions and health during the field Cd stabilization process.

## INTRODUCTION

Due to the intense anthropogenic activities, rapid industrialization, and urbanization, heavy metal contamination in farmland has become a serious threat to food safety and human health (1, 2). Cadmium (Cd) is particularly concerning as a priority pollutant due to its high toxicity in the food web and mobility in crop plants (3, 4). During the field remediation of Cd-contaminated farmlands, the stabilization (or fixation/immobilization) of Cd by soil amendments such as lime (5) and biochar (6) has emerged as a widely used technology because of their low cost and high efficiency. Importantly, restoring soil functions driven by microbiomes to ensure food safety is one of the priority purposes of Cd-contaminated soil remediation.

The rhizosphere of the soybean (*Glycine max* L.) can recruit microbiomes from the bulk soil based on functional traits that promote plant growth (7, 8). By forming the symbiotic nodules with rhizobia, soybean contributes more than 20 million tons of nitrogen (N) annually to agroecosystems (9). Soybean forms obligate symbioses with arbuscular mycorrhizal fungi (AMF), which solubilizes phosphorus (10) and reduces Cd toxicity and uptake (11). Given the benefits of soybean cultivation for improving soil fertility, several studies have explored the potential of legume planting for remediating Cd-contaminated soil (12). Nutrient deficiency is often a hallmark of heavy metal–contaminated soil, making legumes an attractive option for soil remediation (13).

The soil microbiome is composed of complex communities of microorganisms with diverse lifestyles and functions (14). For instance, bacteria and fungi are the most studied groups with crucial functioning in nutrient cycling (15). AMF account for a large proportion of soil microbial biomass and play crucial roles in ecosystem stability and reducing Cd toxicity to host plants (16, 17). Soil fauna, such as protists and nematodes, play vital roles in soil health by predating pathogenic bacteria or fungi (18, 19). Previous studies suggested that nematode predation positively affects nutrient cycling and plant performance (20, 21). Recent studies have demonstrated that these diverse communities are also responsible for soil multifunctions, such as nutrient cycling (22), soil pathogen control (23), and pollutant degradation (24, 25). Interactions among these microbes showed great importance in ecosystem resilience and sustainability (26). There is mounting evidence that the top-down (consumers) and bottom-up (producers) controls among these microbes can strongly influence soil multifunctionality, including nutrient cycling (27, 28), soil biodiversity storage (29), and soil pollutant stresses (30, 31). However, it is unknown whether the effects of soil amendment application on soil multitrophic community structures and functions are positive during Cd-contaminated soil remediation.

To systemically evaluate the ecological restoration effects of soil amendment application on the remediation of Cd-contaminated farmlands, many studies have focused on the changes in bacterial or fungal community diversities and functions (32, 33). Previous field studies showed the important roles of soil amendments in promoting the availability of nutrients, reducing the bioavailability of Cd (34), and altering the structures and functions of soil microbial communities (35). However, these studies often focus on individual microbial communities, neglecting interactions within the soil food web and limiting our understanding of microbiome contributions under Cd stress.

Based on our previous results, higher application of the commercial Mg–Ca–Si conditioner (CMC) performed better in reducing soil Cd bioavailability in the *in situ* remediation of Cd-contaminated soil at two different application rates (36). Thus, we conducted a soybean field assay to examine how soil microbial communities (including groups of soil bacteria, fungi, AMF, and nematodes) are linked to soil functions in the soil samples receiving two different CMC application rates in this study. We also tested soil bacterial ability to inhibit the soilborne pathogen *Fusarium solani* (causing soybean root wilt) (37). Meanwhile, we tested the Cd tolerance of bacterial community suspensions. We hypothesize that the structures and diversities of bulk and rhizosphere microbial communities have a crucial role in regulating soil functions and health under the field Cd stabilization process, and a higher CMC application rate exhibits greater positive effects.

## MATERIALS AND METHODS

### Experiment description and soil sampling

The field experiment was commenced in Cd-contaminated soil in Wenling County (28°21′ N, 121°15′ E, Zhejiang Province, China). The soil is classified as loamy Endoaqualf (38). The physicochemical characteristics of the soil are as follows: pH 5.7, 21.5 mg/kg AP, 32.0 g/kg soil organic carbon, 1.08 mg/kg NH_4_^+^-N, 1.28 mg/kg NO_3_^-^-N, and 1.67 mg/kg total Cd content.

This region has an annual mean temperature of 17.3°C and an annual precipitation of 1,650 mm. The field experiment consisted of three treatments in a randomized block design with three replicate plots (4 m × 5 m for each plot): (i) no amendments (control); (ii) low application rate of CMC (low, 1,500 kg/ha); and (iii) high application rate of CMC (high, 3,000 kg/ha). The CMC is a commercial Mg–Ca–Si conditioner made from carbide slag with pH ranged from 11.0 to 13.0 (Western Environmental Protection Co., Ltd, China). It is composed of 30.0% CaO, 8% MgO, and 4.0% SiO_2_, as described in our previous studies (33, 36).

The soybean cultivar Zhexian-12 (*G. max* L.) is widely grown in Zhejiang Province, China, and was used in this experiment. The field experiment was established under the consecutive application of the soil amendment (CMC) for 2 years in a rice–soybean rotation system. The previous crop was single-cropping late rice, and soybean was sown in April and harvested in June 2021. All soil amendments were applied in late March for soybean and in late July for rice. It was mixed thoroughly by manual plowing to a depth of 0–20 cm. The soybean sowing was conducted 1 week after the stabilization of the soil amendments. For the agronomic measures of all treatments, N fertilizer (urea) was used at a rate of 90 kg/ha and applied before the flowering stage. Irrigation measures are natural rainfall.

Bulk and rhizosphere soil samples from each treatment were collected during the harvest season on 17 June 2021. For the bulk soil sample collection, five randomly selected soil cores (0–20 cm deep and ~20 cm away from the soybean root) from each plot were collected and mixed thoroughly as one composite sample. We randomly selected five soybean plants in each replicate plot for the rhizosphere samples. After shaking off the loosely root-adhered soils, the soil firmly attached to the roots was brushed off and used as rhizosphere soil. Finally, 18 soil samples (2 niches × 3 treatments × 3 replicates) were collected for subsequent analyses.

All the soil samples were transported to the laboratory in an icebox and sifted through a 2-mm sieve to remove the roots and gravel and then homogenized. Half of the samples were stored at −80°C for DNA extraction, and the other samples were stored at 4°C prior to soil physicochemical analyses.

### Measurement of soil physicochemical properties

Soil pH, NH_4_^+^-N, NO_3_^-^-N, SOM, AP, total carbon (TC), total N (TN), total Cd concentrations, and Cd accumulation in soybean were measured according to the methods described in our previous studies (33, 39). Cd speciation was measured using the improved Tessier extraction procedures according to a previous protocol (40), including water-soluble Cd, exchangeable Cd, carbonate bound Cd, iron-manganese oxide bound Cd, organic bound Cd, and residual Cd. The Cd concentrations were measured using inductively coupled plasma mass spectrometry (ICP-MS, PerkinElmer Nexlon300X, USA). The soil-certified reference material GBW07424 (GSS-10) and blanks in batches were employed for quality control, and the recovery rates of Cd for the soil and soybean samples were 97%–108% and 91%–110%, respectively, and the coefficients of variation between the replicates were from 0.2% to 5.4%.

### DNA extraction and high-throughput sequencing

Total genomic DNA was extracted from 0.5 g of fresh soil using FastDNA SPIN kit for soil according to the manufacturer’s instructions (Qbiogene Inc., Carlsbad, CA, USA). DNA quality and concentration were measured using a NanoDrop spectrophotometer (NanoDrop Technologies, Wilmington, DE, USA). The primer pairs used for sequencing were listed in Text S1 in the supplemental material. The DNA quality was checked using agarose gel electrophoresis and Q-bit analysis prior to sequencing. An Illumina Nova6000 platform (Guangdong Magigene Biotechnology Co. Ltd., Guangzhou, China) was used to conduct the high-throughput sequencing of the genomic DNA.

The paired-end sequences were merged and quality-filtered (maximum expected error = 1.0) using USEARCH (v11.0) (41), and the remaining high-quality reads were identified at 100% sequence similarity using *unoise3* (42) with default parameters. Totally, 254,562 bacterial, 155,885 fungal, 71,363 AMF, and 50,169 nematode high-quality sequences were clustered into 17,058 bacterial, 2,950 fungal, 2,659 AMF, and 2,223 nematode zero-radius operational taxonomic units (ZOTUs).

RDP training set v18 was used to assign the taxonomic annotations for bacteria (43). The taxonomic annotation for fungi was performed using the UNITE database (v8.0) (44). AMF sequences were mapped against the MaarjAM AMF database (45), and SILVA (v13.8) database (46) was used to assign taxonomic profiles to phylotypes of nematode. Moreover, nematodes were assigned to trophic groups compared to the proportional representations of bacterivores, fungivores, herbivores, omnivores, and predators (47).

To obtain an equivalent sequencing depth for subsequent microbial community analysis, each microbial ZOTU table was rarefied to the lowest number of sequences (76,169 for bacteria, 78,928 for fungi, 61,971 for AMF, and 60,687 for nematode) before bioinformatics analysis.

### Soil multifunctionality measures

In this study, 23 agroecosystem functions regulated by soil microbiomes were assessed for the quantification of soil multifunctionality: (i) heavy metal control: concentrations of six Cd fractions: water-soluble Cd, exchangeable Cd, carbonate bound Cd, iron-manganese oxide bound Cd, organic bound Cd, and residual Cd; (ii) SOM decomposition: soil enzyme activities related to urea conversion, P mineralization, sugar degradation, and chitin degradation, including urease (SUE), phosphatase (Pho), β-D-glucosidase (BG), and *N*-acetyl-β-glucosaminidase (NAG), which were measured using assay kits purchased from Beijing Solarbio Science & Technology Co., Ltd. Soil basal respiration (SBR) was determined using gas chromatography (GC-2010 Plus SHIMADZU, Japan) after incubation of 5-g fresh soil in a closed 100 cm^3^ soil jar at 25°C; (iii) soil fertility: the available N (ammonium and nitrate) and P; (iv) nutrient cycling: abundances of functional genes involved in carbon cycling (*cbbM*, *chiA*), nitrogen cycling (*nirK*, *nifH*, *amoA-B*, *nirS*), phosphorus cycling (*phoD*), and sulfur cycling (*dsrB*); and (v) pathogen control: the relative abundance of potential fungal plant pathogens was obtained from the sequencing analyses by using FUNguild (48).

Only highly probable and probable guilds were used for subsequent analysis. The inverse abundance (reduced relative abundance) of potential fungal plant pathogens was calculated by (total relative abundance of fungal plant pathogens × −1). The quantitative PCR (qPCR) assays were carried out using the Light Cycler 480 (Roche Applied Science, UK) according to our previous study (49). The mix 20-µL reaction system was composited by 10 µL of SYBR Premix Ex Taq (TaKaRa, Dalian, China), 1 µL of template DNA, 0.5 µL of each primer, and 8 µL of nuclease-free deionized water. The primer sequences and reaction conditions used in qPCR were listed in Table 1. To ameliorate the deviation of the values of functions, we standardized all individual functions to be between 0 and 1 according to the following formula:


$$\frac{F-F_{min}}{F_{max}-F_{min}}$$

where F, F_min_, and F_max_ are the target value, its minimum value, and maximum value, respectively. The multifunctionality was obtained by calculating the average of functions values (50).

**TABLE 1:** Primer sets and programs used in qPCR analysis

Target segment	Primer set	Sequence (5′–3′)	Thermal profile
*amoA-B*	amoA1F	STAATGGTTCTGGCTTAGACG	95°C for 2 min
	amoA2R	GCGGCCATCCATCTGTATGT	40 cycles of 94°C for 20 s, 55°C for 20 s, 72°C for 30 s
*nirK*	FlaCu	ATCATGGTSCTGCCGCG	94°C for 2 min
	R3Cu	GCCTCGATCAGRTTGTGGTT	40 cycles of 94°C for 20 s, 63°C for 30 s, 72°C for 30 s
*nirS*	cd3aF	GTSAACGTSAAGGARACSGG	94°C for 2 min
	R3cd	GASTTCGGRTGSGTCTTGA	40 cycles of 94°C for 45 s, 55°C for 45 s, 72°C for 45 s
*nifH*	PolF	TGCGAYCCSAARGCBGACTC	94°C for 5 min
	PolR	ATSGCCATCATYTCRCCGGA	40 cycles of 95°C for 15 s, 60°C for 30 s, 58°C for 30 s
*phoD*	ALPS-F730	CAGTGGGACGACCACGAGGT	95°C for 2 min
	ALPS-R1101	GAGGCCGATCGGCATGTCG	40 cycles of 95°C for 30 s, 58°C for 30 s, 72°C for 30 s
*cbbM*	cbbM-f	GGCACCATCATCAAGCCCAAG	95°C for 30 s
	cbbM-r	TCTTGCCGTAGCCCATGGTGC	40 cycles of 95°C for 30 s, 57°C for 30 s, 72°C for 20 s
*ChiA*	chif2	GACGGCATCGACATCGATTGG	95°C for 30 s
	chir	CSGTCCAGCCGCGSCCRTA	40 cycles of 95°C for 5 s, 55°C for 30 s, 72°C for 60 s
*DsrB*	DSRp2060F	CAACATCGTYCAYACCCAGGG	95°C for 10 min
	DSR4R	GTGTAGCAGTTACCGCA	40 cycles of 95°C for 40 s, 55°C for 40 s, 72°C for 7 min

### *In vitro* suppressive activities against pathogen *F. solani* and Cd resistance abilities of bacterial suspension

The suppressive activities of soil bacterial community against pathogenic fungi were determined by using *in vitro* dual culture assays. *F. solani* was one of the key soilborne pathogens for soybean, we thereby selected this strain as representative for subsequent experiments. The bacterial suspensions of each treatment were prepared as described by previous studies (51, 52). In brief, 5 g of fresh soil was suspended in a 250-mL Erlenmeyer flask containing 45 mL of sterile phosphate buffer solution. After shaking at 150 rpm at 4°C for 2 hours, we filtered the soil suspension through a 5-µm sterile filter to remove the fungal propagules and generate the “bacteria only” suspension and then it was stored at 4°C (53). We further confirmed the absence of fungi by plating the filtered suspension on potato glucose agar (54). Detailed methods on suppressive activities against pathogen *F. solani* and Cd resistance abilities were described in Text S1 in the supplemental material.

### Statistical analyses

All the statistical analyses and plot graphing in this study were carried out in *R* program (v3.6.3) (https://www.r-project.org). ANOVA and Tukey’s honest significant difference (HSD) test were used to assess the treatment differences in soil physicochemical properties, Cd concentrations in soybean, microbial diversity, trophic groups of nematode community, relative abundances of modules from multitrophic networks, and the suppression effects.

Microbial Shannon indices were calculated based on the rarefied ZOTU tables for each trophic level. The dissimilarity test based on the one-way ANOVA and PERMANOVA was used to compare the community structure of four trophic communities between different treatment groups with *vegan* package (55). The principal coordinate analysis was used to evaluate the Bray–Curtis distances of the bacteria, fungi, AMF, and nematode community compositions in the bulk and rhizosphere soils. Mantel tests were conducted to explore Spearman’s correlations between edaphic properties and the community composition of bacteria, fungi, AMF, and nematode with *vegan* package. Soil multitrophic co-occurrence network was constructed based on the correlation matrix, and the analysis method was described in Text S1 in the supplemental material.

The relationships between soil multifunctionality (standardized average of the function values) and features of each module (relative abundances and number of phylotypes) were tested by ordinary least squares linear regressions to assess the variances in multifunctionality explained (*R* ^2^) by diversity (56). We also performed the spearman correlation analyses between the diversity of each module and a single function and plotted them with *heatmap* package.

In addition, RF model was used to identify the major driving factors of soil multifunctionality with the *randomForest* package. Percent increases in MSE (%IncMSE) of variables were selected to estimate the importance of variables and higher %IncMSE values imply more important variables.

The significance of each predictor was evaluated with 5,000 permutations of the response variable using the “A3” package (57). The relative abundance of each module in treatments was calculated, and biodiversity (species abundance and richness) was counted by the number of phylotypes within each module. SEM was used to evaluate the ecological associations with soil properties (soil pH, NO_3_^-^-N, water-soluble Cd, exchangeable Cd), diversity of keystone module 2 (number of phylotypes of bacteria, fungi, AMF, and nematode within module 2), soil multifunctionality, and soil disease suppression (suppressiveness ratio of bacterial communities on the growth of *F. solani*). The goodness of fit was evaluated by chi-square test, the root mean square error of approximation, and Comparative Fit Index (58). The SEM analyses were conducted using the *lavaan* package (59) in R environment (v3.6.3).

## RESULTS

### Effects of soil amendments on soil properties and Cd accumulation in grains

Our results indicated that Cd concentrations in soybean grains were reduced by 13.8% and 31.3% at the low and high CMC application rate, respectively, compared with the control treatment (Table S1). To determine whether CMC-induced changes in soil properties lead to variations in Cd accumulation in soybean grains, we characterized the edaphic properties of each treatment. Application of CMC significantly altered soil pH (*F*_5,12_ = 5.435, analysis of variance ANOVA, *P* < 0.05) and soil organic matter (*F*_5,12_ = 6.968, ANOVA, *P* < 0.05) compared with the control (Table S2). The available phosphorus (AP) in both bulk and rhizosphere soil significantly increased with low and high CMC application rates (Table S2), with respective increases of 18.1% and 34.4% in bulk soil, and 4.2% and 29.9% in rhizosphere soil, relative to the control.

Although there were no significant differences in total Cd concentrations among the treatments (Table S2), we observed that the Cd fractions (Table 2) and percentages of different forms of Cd were largely altered (Fig. S1A). For instance, the proportion of exchangeable Cd in rhizosphere soil decreased from 21.2% to 17.1% and 13.5%, respectively, while the proportion of iron-manganese oxide bound Cd increased from 14.4% (control) to 20.3% and 21.4%, respectively, in the low and high CMC application rate. Significant correlations between soil pH and Cd fractions, as well as between different Cd fractions and soil properties, were observed (Fig. S1B). For instance, soil organic matter (SOM) showed a strong negative correlation with residual Cd (*P* < 0.05). Our findings showed that CMC amendment application can alter soil pH as well as other physicochemical properties and reduce labile Cd fractions, especially with a high application rate.

**TABLE 2:** Effects of different treatments on Cd fractions (Tukey’s HSD test)^*a*^

Compartment	Bulk soil			Rhizosphere		
Treatment	CK	Low	High	CK	Low	High
Water-soluble Cd (mg/kg)	0.022 ± 0.001a	0.022 ± 0.001a	0.022 ± 0.000a	0.024 ± 0.000a	0.023 ± 0.000a	0.022 ± 0.003a
Exchangeable Cd (mg/kg)	0.31 ± 0.03a	0.26 ± 0.12a	0.24 ± 0.03a	0.36 ± 0.11a	0.31 ± 0.13a	0.40 ± 0.13a
Carbonate bound Cd (mg/kg)	0.19 ± 0.03ab	0.27 ± 0.15b	0.36 ± 0.03a	0.19 ± 0.01b	0.21 ± 0.08b	0.21 ± 0.03b
Iron-manganese oxide bound Cd (mg/kg)	0.25 ± 0.02a	0.32 ± 0.13a	0.39 ± 0.05a	0.25 ± 0.00a	0.36 ± 0.10a	0.36 ± 0.08a
Organic bound Cd (mg/kg)	0.06 ± 0.00ab	0.06 ± 0.01ab	0.07 ± 0.01a	0.06 ± 0.00b	0.06 ± 0.01ab	0.06 ± 0.01ab
Residual Cd (mg/kg)	0.85 ± 0.08a	0.85 ± 0.13a	0.90 ± 0.12a	0.73 ± 0.10a	0.79 ± 0.11a	0.64 ± 0.40a
Total Cd (mg/kg)	1.67 ± 0.16a	1.69 ± 0.07a	1.70 ± 0.17a	1.66 ± 0.10a	1.75 ± 0.34a	1.67 ± 0.24a

^*a*^ Different letters mean significant differences between samples within rows (*P* < 0.05).

### Diversity and assembly of four microbial communities

**Fig 1:**
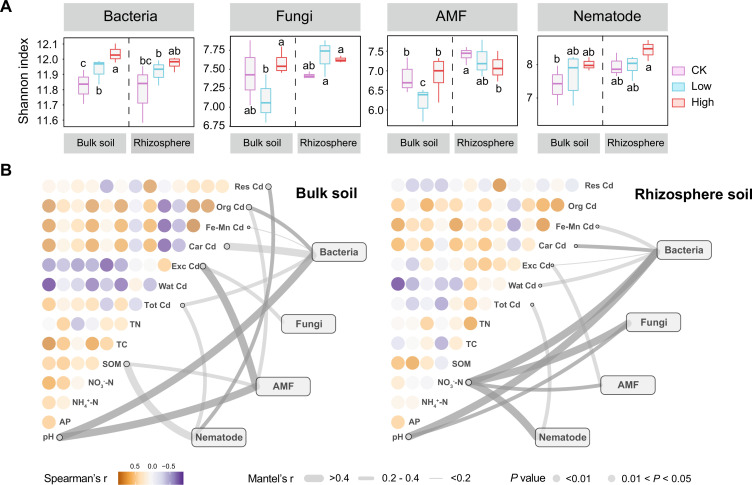
Responses of multitrophic communities to different treatments in the bulk and rhizosphere soils. (**A**) Microbial alpha diversity of different trophic communities in the bulk and rhizosphere soils. Significant comparisons between treatments are indicated by different letters as defined by Tukey’s HSD test. (**B**) Correlations of the four trophic communities with edaphic properties. Edge width represents the Mantel’s *r* value, and the edge color denotes the statistical significance. Gradient gray color denotes Spearman correlation coefficients. The abbreviations in the figure are consistent with Fig. S1.

Our results indicated that the application of CMC significantly increased microbial Shannon index in both bulk soil and rhizosphere soil (Fig. 1A). Except for the AMF communities, the Shannon diversities of the bacterial, fungal, and nematode communities were highest in the treatment of high CMC application rate. In contrast, the Shannon diversity of the AMF community showed an opposite trend. Analysis of similarities (ANOSIM) and permutational multivariate analysis of variance (PERMANOVA) revealed that the niche compartment and treatment significantly affected the microbial community compositions including fungi, AMF, and nematode (Table S3 and Fig. S2). However, no significant effects were observed for the bacteria community (Table S3 and Fig. S2A). Taxonomic classification of the bacterial microbes in the bulk and rhizosphere soils assigned most of them to Proteobacteria (40.1%), Acidobacteria (13.9%), Actinobacteria (12.3%), and Bacteroidetes (5.8%) at the phylum level (Fig. S2A). Fungal community was dominated by Sordariomycetes (62.3%), Dothideomycetes (16.2%), and Agaricomycetes (8.1%) in the bulk and rhizosphere soils (Fig. S2B). As for the AMF community composition, the dominant genera were *Paraglomus* (27.5%), *Glomus* (27.0%), and *Gigaspora* (21.6%) (Fig. S2C). The dominant nematodes in soils were *Rhabditidae* (41.1%), *Onchocercidae* (11.2%), and the fungivorous nematode genus *Aphelenchoididae* (8.1%) (Fig. S2D). We further assigned the nematodes to different trophic groups based on a previous study (47). In our research, bacterivores accounted for the vast majority of the nematode communities, followed by herbivores and fungivores (Fig. S3). Treatments with CMC significantly increased the relative abundances of fungivores (*P* < 0.05) and omnivores (*P* < 0.05) in the rhizosphere and bulk soil, respectively (Fig. S3B and D). Neither bacterivores nor herbivores differed significantly among the treatments (Fig. S3A and C). Moreover, predators were sensitive to CMC application, as evidenced by drastic changes in their relative abundances (Fig. S3E).

The Mantel test further showed that the structures of the four microbial communities were significantly and strongly correlated with properties, such as pH, nitrate N (NO_3_^-^-N), SOM, and different Cd fractions of the bulk and rhizosphere soils (Fig. 1B). At the trophic level, the relationships between bacterial community and Cd fractions were more significant than those in other microbial communities. Among various edaphic features, soil pH and Cd fractions (exchangeable Cd and carbonate bound Cd) had the most obvious effects on microbial communities in both bulk and rhizosphere soils.

We also found the significant effects of SOM on the nematode and AMF communities in the bulk soil, and strong associations between NO_3_^-^-N concentration and the four microbial communities in the rhizosphere soil. The above observations indicate that CMC had a significant impact on the assemblages of soil microbial communities by affecting edaphic characteristics.

### Keystone multitrophic module relating to multifunctionality

**Fig 2:**
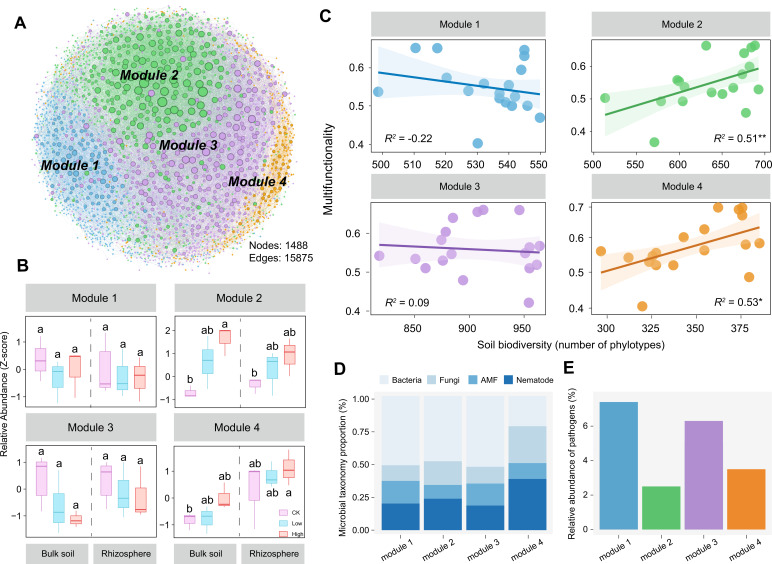
Relationships between the biodiversity of phylotypes within modules of multitrophic co-occurrence network and the multifunctionality. (**A**) Multitrophic network with colored nodes denoting four main modules. The size of each node is proportional to the relative abundance of the ZOTUs. (**B**) Relative abundances of the modules in bulk and rhizosphere soils. Different letters indicate significant difference between treatments as defined by Tukey’s HSD test. (**C**) The linear relationships between multifunctionality and the biodiversity (number of phylotypes) of phylotypes within the network modules. The bright colorful lines represent the significant linear correlations, and the shaded areas indicate the 95% confidence interval of the fit. *P* values were indicated by asterisks: *P < 0.05, **P < 0.01. (**D**) Microbial taxonomy proportion of the dominant phylotypes in the modules. (**E**) Relative abundance of potential plant pathogens in each module.

Based on the observed changes in microbial community compositions, we constructed a multitrophic co-occurrence network at the phylotype level to further explore the associations (e.g., ecological modules) among different microbial lineage taxa and their contributions to soil multifunctionality. A multitrophic network of 1,488 nodes and 15,875 edges was constructed and four major modules were identified from the network (Fig. 2A). It was observed that the relative abundances of modules 2 and 4 increased with higher CMC application rate (Fig. 2B). Specifically, the biodiversity (richness of phylotypes of bacteria, fungi, AMF, and nematode within the module) and relative abundance of module 2 showed significantly positive correlations with the soil multifunctionality (Fig. 2C; Fig. S4A).

**Fig 3:**
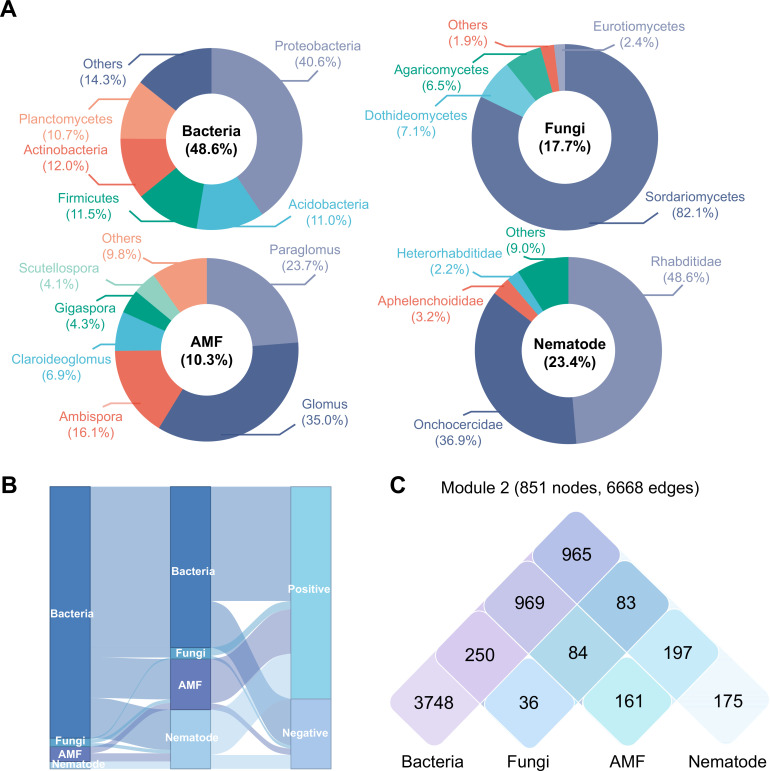
The multitrophic community assemblage of the phylotypes within keystone module 2. (**A**) Relative abundances of microbial compositions of module 2. (**B**) Interactions between different trophic microbes within module 2. (**C**) Venn diagram of the number of unique and shared interactions between the trophic microbes in module 2.

We also found that the proportions of relative abundance at the different phylotype levels in module 2 were dominated by bacteria community (48.6%), followed by nematode (23.4%), fungi (17.7%), and AMF (10.3%) (Fig. 2D and Fig. 3). Also, we calculated the relative abundances of potential fungal pathogens inferred by FUNguild and found that the relative abundances of potential fungal pathogens were lowest in module 2 (Fig. 2E). Given the above critical role of module 2 in ecological functions, we refer module 2 as the keystone module in the following analysis.

Further detailed analysis of microbial community composition indicated that module 2 contained diverse microbial phylotypes (Fig. 3A). The interactions within module 2 were dominated by bacteria–bacteria (Fig. 3B). Additionally, there were more negative correlations among phylotypes within module 2 (24.8%, Fig. 3B), compared with other modules (12.2%, 18.6%, and 10.8% in module 1, module 3, and module 4, respectively, Fig. S4). In comparison, module 2 contained most of the nodes and edges of the multitrophic networks, and the phylotypes of bacteria were in dominance (Fig. 3C; Fig. S4B). Collectively, the above data suggest that applying a higher amount of CMC can increase the relative abundances and biodiversity of keystone module within the multitrophic networks, as well as the interactions among phylotypes at various trophic levels, which are closely related to the soil multifunctionality.

### Main drivers of the soil multifunctionality

**Fig 4:**
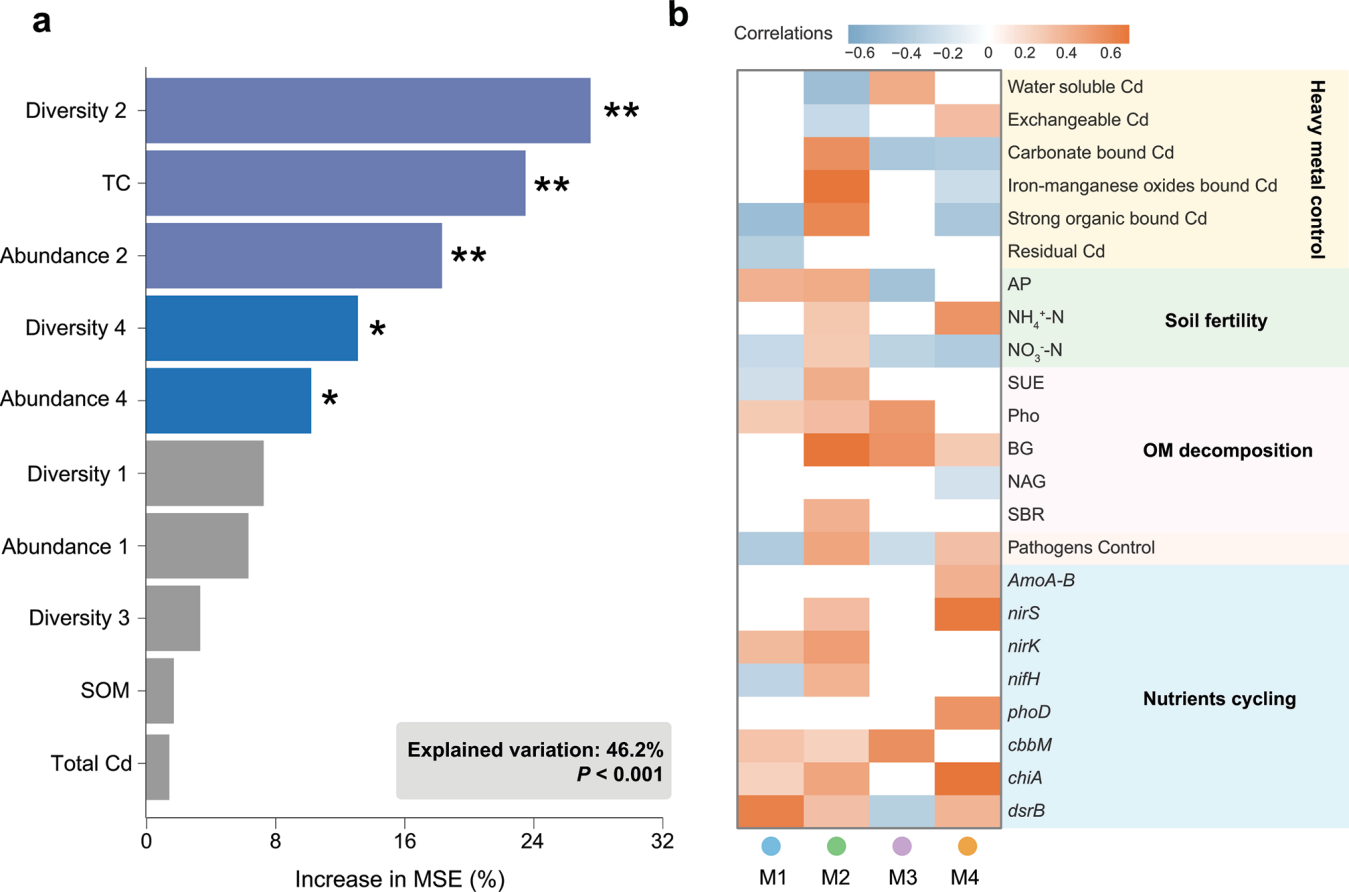
The drivers of the multifunctionality of soil microbiome and the links between the multitrophic co-occurrence network and multifunctionality. (a) Mean predictor importance (increase in MSE%; percent increase in mean square error) of influencing factors as explanatory variables for the multifunctionality. The higher MSE% values represent more important predictors. Abundance 1–4, the relative abundance of modules 1–4; Diversity 1–4, the richness of phylotypes within modules 1–4; SOM, soil organic carbon; TC, total carbon. Significant levels of each factor are *P < 0.05, **P < 0.01. (b) Spearman correlations between the diversity of each module and soil multifunctionality. Only the significant (*P* < 0.05) correlations are shown.

Next, we sought to identify the main ecological drivers of soil multifunctionality using random forest (RF) model analysis. The RF model we built explained 46.2% of the variations in soil multifunctionality and indicated that the biodiversity and relative abundances of dominant phylotypes within module 2 were the significant and important predictors for soil multifunctionality (Fig. 4). Further correlation analysis showed that the richness of phylotypes within keystone module 2 had significantly positive associations with the greatest number of a single function (Fig. 4B). For instance, the diversity of module 2 was consistently and positively correlated with various functions related to heavy metal contents (e.g., bound-form Cd), soil fertility (e.g., AP, ammonia N [NH_4_^+^-N], and NO_3_^-^-N), organic matter decomposition (e.g., SUE, Pho, BG, and SBR), pathogens control, and nutrient cycling (e.g., N cycling genes, C cycling genes, and S cycling genes). Overall, our findings showed that a higher CMC application rate promotes the diversity of the keystone module 2, which is critical in sustaining soil multifunctionality and enhancing soil carbon storage.

### Inhibition of the soybean pathogen *F. solani* by the soil bacterial community *in vitro*

The above findings revealed that module 2 was characterized by the lowest potential fungal pathogens, dominated by bacteria, and the relative abundance of module 2 was higher in treatments with CMC application compared with the control (Fig. 2). Meanwhile, the relative abundance and biodiversity of module 2 were the vital biotic factors influencing multifunctionality (Fig. 2 and(Fig. 4) ). Given the observed important roles of the bacterial community of module 2, we therefore focused subsequent analyses on the bacterial community. The bacterial suspensions of each treatment were then tested in dual co-culture assays for their antagonism against the pathogenic fungus *F. solani*.

**Fig 5:**
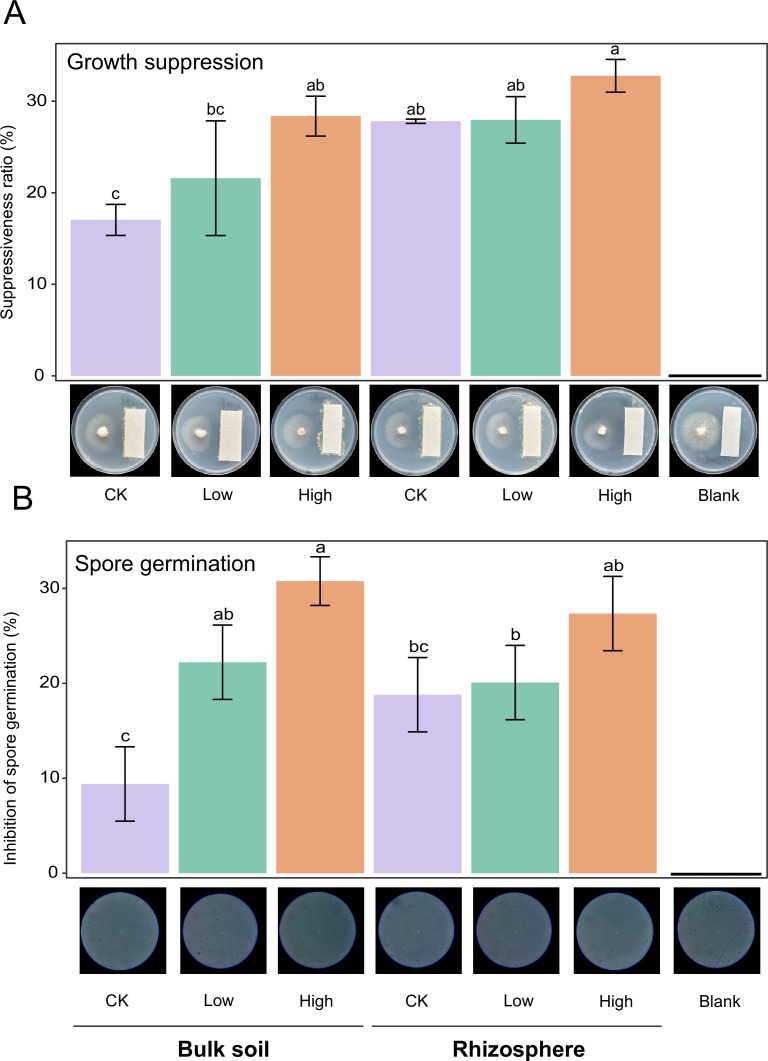
Pathogen-suppressive abilities of bacterial communities derived from different treatments on the *Fusarium solani* growth. (**A**) Effect of bacterial suspensions on *F. solani* growth suppression. (**B**) Effect of bacterial suspensions on spore germination. In panels A and B, different letters indicate statistically significant differences between treatments as determined by Tukey’s HSD test (*P* < 0.05).

In comparison with the control suspensions, bacterial suspensions obtained from the CMC treatments revealed substantial antagonistic activities in mycelium growth in both the bulk and rhizosphere soil samples (*F*_5,12_ = 10.09, ANOVA, *P* < 0.001; Fig. 5A). Further, bacterial communities in the high CMC application rate treatment showed the greatest growth suppression effects, with percent inhibition of growth values being 28.4 ± 2.2% and 32.8 ± 1.8%, respectively, in the bulk and rhizosphere soils (Fig. 5A).

Similarly, we observed that bacterial communities in the CMC treatments can strongly suppress fungal spore germination (*F*_5,12_ = 11.94, ANOVA, *P* < 0.001; Fig. 5B). We further found that bacterial suspensions produced from high CMC application treatments could significantly reduce spore germination, with percent suppression of spore germination of 61.5 ± 5.1% and 54.7 ± 7.8%, respectively, in the bulk and rhizosphere soils (Fig. 5B). It is notable that rhizosphere soil showed greater pathogen growth inhibition, while bulk soil exhibited stronger spore germination reduction.

### Assessment of Cd resistance of soil bacterial community *in vitro*

To test whether the application of CMC could enhance soil bacterial community’s resistance to Cd, we determined the minimum inhibitory concentrations (MICs) of soil bacterial suspensions and compared the MIC of different treatments. We found that bacterial communities derived from CMC-amended soils had higher Cd resistance than that of the control treatment, and such resistance increased as the CMC application rate increased (Fig. S5). Notably, the bacterial suspensions in the bulk soil exhibited the maximum Cd resistance at 0.8 mM (*F*_2,6_ = 3.67, ANOVA, *P* < 0.05; Fig. S5), but the highest Cd resistance in the rhizosphere soil is at concentrations of 0.4 (*F*_2,6_ = 4.186, ANOVA, *P* < 0.05; Fig. S5) and 0.6 mM (*F*_2,6_ = 18.84, ANOVA, *P* < 0.01; Fig. S5). These results indicate that the application of CMC can enhance the bacterial community’s resistance to Cd during Cd-contaminated soil restoration.

## DISCUSSION

In this study, we compared the effects of two application rates of a soil amendment (CMC) on edaphic properties and microbial communities and functions in a Cd-contaminated soybean field. We demonstrated that the biodiversity of microbes within the keystone module is critical for maintaining numerous ecosystem processes that are related to heavy metal control, SOM decomposition, soil fertility, nutrient cycling, and pathogen control. Notably, CMC application showed a great potential in sustaining soil health by inhibiting *F. solani* mycelium growth and spore germination. In conclusion, the application of soil amendments such as CMC, particularly in large doses, can increase the relative abundance of keystone microbial modules. These modules are crucially linked to the multifunctionality of agroecosystems and the control of diseases during the remediation of Cd-contaminated soils.

### Soil amendments altered soil pH and Cd speciation and regulated soil microbial communities

In agricultural soil, Cd speciation determines biotoxicity more than Cd concentration (60). Most of the previous studies focused on the labile Cd fractions as they are more soluble and bioavailable than the stable forms (32, 61). Here, we investigated the changes in different Cd speciation and edaphic physicochemical properties under CMC application. We found that applying CMC increased stable Cd fractions (e.g., iron-manganese oxide bound Cd and strong organic bound Cd) and decreased labile Cd fractions (e.g., water-soluble Cd and exchangeable Cd) (Fig. S1A). Notably, treatments with higher amounts of CMC had better performance in Cd immobilization, which is attributed to the increased soil pH resulting from higher CMC application (39). Spearman correlation analysis also revealed that Cd speciation is mainly controlled by soil pH. For instance, the concentration of water-soluble Cd is negatively correlated with soil pH (*P* < 0.01; Fig. S1B), which is consistent with previous studies (32, 62). We found positive correlations between AP and soil pH, and between NO_3_^-^-N and carbonate/iron-manganese oxide bound Cd (Fig. S1B), indicating that the CMC soil amendment can reduce Cd availability while enhancing soil N and phosphorus (P) nutrients (33, 39). N and P are essential nutritional elements that can dramatically boost soybean plant biomass and growth (63). Under the circumstances of Cd stress, they can also improve the tolerance of plants by increasing the activity of antioxidant enzymes and therefore reducing Cd’s toxicity (64, 65). Moreover, the relative abundances of genes involved in N (e.g., *nirS*, *nifH*) and P (*phoD*) cycling showed significant enrichment in rhizosphere soil and CMC-amended soils (Table S4), indicating the improvement of soil functions (33).

The results based on our microbial analysis illustrate that CMC application increased the alpha diversity for bacteria, fungi, and nematode but reduced the alpha diversity of the AMF community (Fig. 1A). This pattern can be explained by the fact that CMC improved soil nutrients, especially soil N and P. Low concentrations of AP can promote the diversity of AMF, while high concentrations of AP may inhibit the growth and development of AMF community (66). A similar phenomenon was also found in a long-term field fertilization experiment indicating that organic fertilization decreased AMF Shannon and Chao1 diversity (27). Additionally, the application of CMC can also affect the multitrophic community assemblies by affecting the soil chemical properties, mainly including soil pH, NO_3_^-^-N, SOM, and Cd speciation (exchangeable Cd, carbonate bound Cd, and iron-manganese oxide bound Cd) (Fig. 1B). We found that soil phylotypes with larger sizes or at higher trophic levels such as fungi and nematode appeared to be more sensitive to soil nutrients. Consistent with previous studies, larger microbial taxa are more influenced by deterministic processes, indicating greater responsiveness of microbial communities to environmental changes (67). Meanwhile, predation by larger taxa (e.g., nematodes) on smaller taxa (e.g., bacteria) plays a crucial role in promoting plant growth and health under stress (68). Thus, we conclude that soil amendment–induced changes in edaphic environments can influence various microbial communities, and trophic interactions could also shape different microbial groups to enhance microbial functions.

### Keystone module largely contributed to soil multifunctionality

Deciphering the diversity of root-associated microbiomes of soybean and their complex relationships is of great importance in utilizing microbiota to improve soybean growth under heavy metal stresses (69). The rhizosphere microbial community assembled by soybean have some specific metabolic pathways and functions (7). Our results showed that the contents of nutrients (SOM and NH_4_^+^-N) and relative abundances of functional genes were higher in rhizosphere soil than those in bulk soil (Table S4). In particular, the relative abundance and biodiversity of the respective keystone phylotypes within the multitrophic module were positively correlated with the soil multifunctionality at a high significant level (*P* < 0.01). Soil biota is considered as an important component of the soil food webs (26), and its diversity is vital for the numerous ecosystem functions. Consistent with recent studies (50, 56, 70), our results provided robust evidence to support the critical roles of keystone phylotypes and their diversities to maintain multiple functions (Fig. 4A). Amelioration of Cd-contaminated soil enhanced the interactions between soil microbes, consistent with previous studies showing that applying soil amendments promoted the recovery of rare bacterial and fungal taxa associated with soil multifunctionality (35). Thus, the functioning of the microbial community is suitable to be used as an indicator to assess the efficiency of *in situ* restoration strategies (71). To the best of our knowledge, this is the first study to link soil trophic microbial communities to soil multifunctionality in Cd-contaminated soil remediation. Considering the importance of species within multitrophic modules for maintaining soil functions, this approach may have great potential for applying soil amendment to restore agroecosystem multifunctionality in Cd-contaminated soils.

Based on the multitrophic network analysis, most of the interactions within keystone module 2 dominated by bacteria–bacteria, bacteria–fungi, and AMF–nematode were negatively correlated (Fig. 3). The importance of negative interactions between microorganisms for community functioning has been widely investigated (72, 73). One recent study has demonstrated that negative correlations represent competition between species for limited nutrient resources (74). The more negative values represent the more stable microbial communities (75). Our findings indicated that the keystone module 2 had more negative correlations among phylotypes compared with other modules (Fig. 3B), suggesting multitrophic communities in module 2 are more stable (72). However, the exact mechanisms underlying these competition relations are unknown and need to be further investigated. Among the lineages shared within microbial communities in module 2, we found that there existed groups known as plant growth–promoting phylotypes such as bacterial phylotypes of Proteobacteria (*Aggregicoccus* [Myxococcaceae family] and *Sphingomonas* [Sphingomonadaceae family]) (Table S5). These phylotypes can colonize the rhizosphere of hyperaccumulators to mobilize Cd (76). Also, Isosphaeraceae (Planctomycetales order) and *Paenibacillus* (Paenibacillaceae family) are also key taxa in module 2 and closely associated with nutrient cycling and plant growth promoting (77, 78). Moreover, Sordariomycetes and Dothideomycetes in module 2 are two common fungal communities. Previous studies have shown that these two fungal taxa are more resistant to Cd stress due to their broader environmental tolerance and greater range of functions that help maintain agroecosystem functioning (35). AMF have been shown to enhance nutrient uptake and activate the enzymatic defense system of host plants in response to heavy metal stress (79 - 81). For instance, the presence of *Glomus versiforme* significantly decreased the Cd accumulation in shoots and induced the improvement of catalase, guaiacol peroxidase, and ascorbate peroxidase activities to relieve Cd phytotoxicity (82). For the nematode community, *Bursaphelenchus* (Aphelenchoididae family) is a common fungal-feeding nematode that plays an important role in regulating nutrient cycling and plant growth by preying on fungi (27, 83).

### Soil amendments promoted disease suppression and Cd resistance of bacterial communities

Healthy soils are the basis for healthy food production to feed people and animals (84, 85). Application of soil amendments has been proven to be able to support soil microbiome for soil health. For instance, acidic soil amelioration by organic amendments can enhance the antagonistic effects of rhizosphere bacterial communities by enriching some beneficial microbial species such as *Sphingomonas* in Proteobacteria and *Nocardioides* in Actinobacteria (51). A recent study revealed that organic fertilizer can improve plant health by promoting beneficial interactions between protist Cercozoa and bacteria *Bacillus* that suppress the growth of harmful *Fusarium oxysporum* fungi (68).

Recent research has found that in amendment-treated Cd-contaminated soil, plant pathogenic fungi such as *Fusarium* and *Alternaria* are reduced, while ecologically beneficial organisms such as *Nitrospira* and *Bacillus* are abundant (86). We showed through *in vitro* assays that using bacterial suspensions from treated amendments significantly improves pathogen defense by inhibiting the growth and germination of fungal spores (Fig. 5). We also verified that CMC application could enhance the resistance of the bacterial community to Cd stress (Fig. 5). Similar observations have been documented in Cd-contaminated paddy soils where the relative abundances of heavy metal–resistant bacteria significantly increased with titanium gypsum amendment (87).

**Fig 6:**
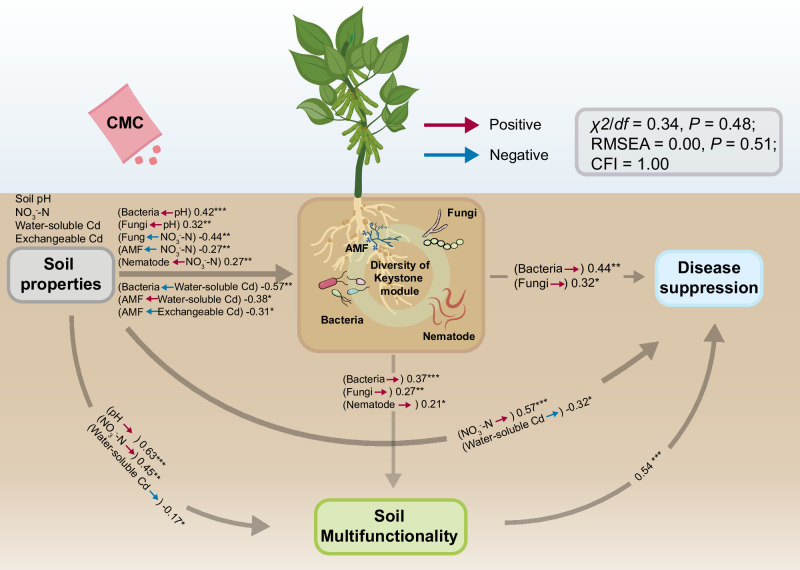
Structural equation model (SEM) describing the ecological associations in soil properties (soil pH, NO_3_^-^-N, water-soluble Cd, exchangeable Cd), diversity of keystone module 2 (number of phylotypes of bacteria, fungi, AMF, and nematode within module 2), soil multifunctionality, and soil disease suppression (suppressiveness ratio of bacterial communities on the growth of *Fusarium solani*). Red arrows represent positive pathways, and blue arrows represent negative pathways. The proportion of variance explained is indicated by *R* ^2^. The goodness-of-fit statistics for each model are *χ* ^2^, chi-square test; *df*, degrees of freedom; *P*, probability level; RMSEA, root mean square error of approximation. *P < 0.05, **P < 0.01, ***P < 0.001 are the significance values for each predictor.

We further conducted the structural equation model (SEM) analysis to test the multiple relationships between soil properties, keystone module, soil multifunctionality, and disease suppression (Fig. 6). Given that the microbial communities were significantly associated with soil pH and NO_3_^-^-N, and the water-soluble Cd and exchangeable Cd were the labile Cd fractions and showed a gradual decrease with CMC application, we selected these four factors as soil properties used in SEM. Our results indicated the diversity of phylotypes within the keystone module was strongly linked to soil disease suppression, and these relationships were associated with soil properties and functioning (Fig. 6). The diversity of keystone microbial groups is all known to regulate the pivotal process in diverse ecosystems (88, 89). In this study, we highlight the positive effects of using CMC at two rates on microbial community structures, as well as the fundamental role of phylotypes within keystone multitrophic modules in driving soil multifunctionality and maintaining soil health. We provided solid evidence that the diversity of microbial communities at different trophic levels is of great importance in the recovery of multifunctionality and sustainment of soil health, especially at the higher application rate. We also identified a list of potential microbial species within keystone modules such as *Aggregicoccus* (bacteria), Sordariomycetes (fungi), *Glomus* (AMF), and *Bursaphelenchus* (nematode) (Table S5) that were strongly linked to soil multifunctionality. Our findings open the possibility of improving the restoration effects of Cd-contaminated soils by mediating these species.

## Data Availability

The raw sequencing data have been submitted (PRJCA005884) in the Genome Sequence Archive in the BIG Data Center, Chinese Academy of Sciences (http://bigd.big.ac.cn/gsa), with the accession numbers CRA007026 for bacteria, CRA007027 for fungi, CRA007028 for AMF, and CRA007029 for nematode.

## SUPPLEMENTAL MATERIAL

FIG S1 msystems.01049-22-s0001.eps (A) Effects of different treatments on the percentages of distinct forms of Cd in total Cd. The number on pieces of sector charts represent the percentage of different forms of Cd in the total Cd. (B) Spearman correlation between soil properties. Asterisks indicate significant correlations (* P < 0.05, ** P < 0.01). Tot Cd, total Cd; Wat Cd, water soluble Cd; Exc Cd, exchangeable Cd; Car Cd, carbonate bound Cd; Fe-Mn Cd, iron-manganese oxides bound Cd; Org Cd, organic bound Cd; Res Cd, residual Cd.

FIG S2 msystems.01049-22-s0002.eps The diversity and composition of microbial communities in bulk and rhizosphere soil for bacteria (A), fungi (B), AMF (C), and nematode (D). PCoA of all treatments of bulk and rhizosphere soil based on Bray-Curtis distance, respectively. Taxonomic classification of the ZOTUs of bacteria, fungi, AMF and nematode were performed at the phylum level, class level, genus level and family level, respectively.

FIG S3 msystems.01049-22-s0003.eps The trophic groups of nematode community. (a-e) Relative abundances of trophic groups of nematode community. Different letters indicate statistical significance (P < 0.05) as Tukey’s HSD test.

FIG S4 msystems.01049-22-s0004.eps (A) The linear relationships between the relative abundance of modules and soil multifunctionality. (B) The trophic interactions between different microbes within module 1, 3, and 4. Venn diagram of the number of unique and shared interactions between the trophic microbes in module 1, 3, and 4.

FIG S5 msystems.01049-22-s0005.eps Determination of cadmium resistance of bacterial suspensions of different treatments. Asterisks indicate statistical significance (P < 0.05) as Tukey’s HSD test.

TABLE S1 msystems.01049-22-s0006.csv Cd concentrations in grains and decreasing rates of the Cd concentrations in soybean grains.

TABLE S2 msystems.01049-22-s0007.csv Effects of different treatments on soil properties (Tukey’s HSD test).

TABLE S3 msystems.01049-22-s0008.csv Effects of compartment and soil amendments treatments on the microbial community base on ANOSIM and PERMANOVA.

TABLE S4 msystems.01049-22-s0009.csv Difference in relative abundances of functional genes.

TABLE S5 msystems.01049-22-s0010.csv Taxonomic information of key taxa in module 2.

SUPPLEMENTAL TEXT S1 msystems.01049-22-s0011.docx Methods about 16S sequencing, pathogen growth inhibition assays, spore germination inhibition assays, Cd resistance abilities assays, and soil multitrophic co-occurrence networks.

ASM does not own the copyrights to Supplemental Material that may be linked to, or accessed through, an article. The authors have granted ASM a non-exclusive, world-wide license to publish the Supplemental Material files. Please contact the corresponding author directly for reuse.
