# Supplementary material for: Enhanced soil function and health by soybean root microbial communities during in situ remediation of Cd-contaminated soil with the application of soil amendments
Source: mSystems. 2023 May 18;8(3):e01049-22. doi: 10.1128/msystems.01049-22 (PMC10308949; doi:10.1128/msystems.01049-22)
Supplement: SUPPLEMENTAL TEXT S1 — Methods about 16S sequencing, pathogen growth inhibition assays, spore germination inhibition assays, Cd resistance abilities assays, and soil multitrophic co-occurrence networks. [file msystems.01049-22-s0011.docx]

**Supplemental Text S1**

**16S sequencing:**

Bacterial 16S rRNA genes was amplified using primer pair 515F (5’-GTGCCA GCMGCCGCGGTAA-3’) and 806R(5’-GGACTACHVGGGTWTCTAAT-3’) (1). Fungal ITS2 region was amplified using primer pair fITS7 (5’-GTGARTCATCGAATCTTTG-3’) and ITS4 (5’-TCCTCCGCTTATTGATATGC-3’) (2). Primer pair AMV4.5NF (5’-AAGCTCGTAGTTGAATTTCG-3’) and AMDGR (5’-CCCAACTATCCCTATTAATCAT-3’) (3)was used for amplifying soil 18S rRNA gene fragments for the arbuscular mycorrhizal fungi (AMF) region. Primer pair NF-1 (5’-GGTGGTGCATGGCCGTTCTTAGTT-3’) and 18Sr2b (5’-TACAAAGGGCAGGGACGTAAT-3’) (4) was used for amplifying the Nematode 18S genes.

**Pathogen growth inhibition assays:**

The 50 μl of bacterial suspension was evenly applied on a sterile cellulose filter (2 cm × 5 cm) that placed on one side of the 1/10 strength TSB agar and then sealed with Parafilm and incubated at 25 ^°^C for 7 days (5). After incubation, a 5-mm diameter mycelial plug of *F. solani* (pre-cultured on PDA medium at 25 ^°^C) was placed on the opposite side of bacterial inoculation. Plates inoculated with *F. solani* and an equal volume of sterile deionized water only served as blank control, and four replicate dishes for each blank control and treatment were performed. Following 3 days incubation at 25 ^°^C, the growth suppressiveness ratio (%) was calculated as follows: 100 × [(Ga−Gp)/Ga], where Ga was the radial growth of *F. solani* in the blank control and Gp was the radial growth of *F. solani* in the dual culture assays.

**Spore germination inhibition assays:**

Pathogenic fungi suspension were collected from the 4-day-old PDA medium incubated at 25 °C by centrifugation for 10 min at 10,000 ×g. The spore suspension was adjusted to a final concentration of 1 × 10^5^ cells per ml by diluting in sterile deionized water. And then, we mixed 270 μl of bacterial suspension and 30 μl of the spore suspension in the well of sterile 96-well plate. Spore germination rates (%) was evaluated following 12h incubation at 25 °C. The spore was considered as germinated if the length of germ tube exceeds the length of spore. The spore suspension mixed with 270 μl of sterile deionized water served as blank controls, and three replicates for each blank control and treatment were performed. The percent of spore germination inhibition was calculated using the following formula: (G1−G2)/G1, where G1 represents the percentage of the blank control spore germination and G2 represents the percentage of bacterial suspension spore germination (6).

**Cd resistance abilities assays:**

The lowest concentration of Cd in culture medium that completely inhibit the visible growth of bacteria is defined as Minimum inhibitory concentration (MIC), which could reflect the resistance of bacteria to Cd (7, 8). We tested the OD_600_ value of bacterial suspension before culture to ensure consistency of the concentration of bacterial suspension in each treatment, and no significant differences were observed. Thus, we assumed that the bacterial suspension concentration in each treatment is the same before culture. For the assay, 10 μl of bacterial suspension was transferred into sterile 96-deep-well plates (each well contained 1,000 μl of 1/10 strength liquid TSB medium) that containing gradient concentrations of 0, 0.1, 0.2, 0.4, 0.6, 0.8, 1.0, 1.2, and 1.5 mM CdCl_2_. For each sample, three replicate wells were analyzed. After incubation in a horizontal shaker at 28 °C for 24 h, the absorbance value of bacterial solution at 600 nm was measured (OD_600_). Taking Cd^2+^ concentration as abscissa and OD_600_ as ordinate, we plotted the MIC curves with different Cd^2+^ concentrations, and Cd resistance capacity of each bacterial suspension was assessed.

**Soil multitrophic co-occurrence networks:**

The soil multitrophic network was constructed based on a significant spearman correlation matrix. Only abundant microorganism (ZOTUs) with a relative abundance more than 0.01% and sum accounting for >75% of the total relative abundance for bacteria, fungi, AMF, and nematode communities were selected for the construction of networks (9). In total, 1749 bacterial ZOTUs, 707 fungal ZOTUs, 653 AMF ZOTUs, and 1106 nematode ZOTUs were obtained, and then we merged the filtered ZOTU tables into an abundance table (10). All Spearman’s correlation coefficients were calculated with the *corr.test* function in the *psych* package and *P* values were adjusted by Benjamini-Hochberg’s FDR method (11), and the cutoff of FDR adjusted *P* values was 0.01. The threshold value of correlation coefficients was performed as 0.8 (12). Finally, the meta-community co-occurrence network consisted of 1488 nodes and 15,875 edges. Network properties (number of nodes and edges, modularity, average clustering coefficient, and average path length) were calculated using the *igraph* package (13). Gephi platform (v0.9.2) (14) was used to visualize the network based on the Fruchterman-Reingold algorithm and identify the modules where soil microbes strongly interacting with each other. Meanwhile, the relative abundance of each module in different treatments was calculated, and biodiversity (species abundance and richness) was counted by the number of phylotypes within each module.

**REFERENCES:**

1. Feng J, Shen X, Chen J, Shi J, Xu J, Tang C, Brookes PC, He Y. 2019. Improved rhizoremediation for decabromodiphenyl ether (BDE-209) in E-waste contaminated soils. Soil Ecology Letters 1:157–173.

2. Ihrmark K, Bödeker ITM, Cruz-Martinez K, Friberg H, Kubartova A, Schenck J, Strid Y, Stenlid J, Brandström-Durling M, Clemmensen KE, Lindahl BD. 2012. New primers to amplify the fungal ITS2 region – evaluation by 454-sequencing of artificial and natural communities. FEMS Microbiology Ecology 82:666–677.

3. Lumini E, Orgiazzi A, Borriello R, Bonfante P, Bianciotto V. 2010. Disclosing arbuscular mycorrhizal fungal biodiversity in soil through a land-use gradient using a pyrosequencing approach. Environmental Microbiology 12:2165–2179.

4. Porazinska DL, Giblin-Davis RM, Faller L, Farmerie W, Kanzaki N, Morris K, Powers TO, Tucker AE, Sung W, Thomas WK. 2009. Evaluating high-throughput sequencing as a method for metagenomic analysis of nematode diversity. Molecular Ecology Resources 9:1439–1450.

5. de Boer W, Wagenaar A-M, Klein Gunnewiek PJA, van Veen JA. 2007. In vitro suppression of fungi caused by combinations of apparently non-antagonistic soil bacteria. FEMS Microbiology Ecology 59:177–185.

6. Li P-D, Zhu Z-R, Zhang Y, Xu J, Wang H, Wang Z, Li H. 2022. The phyllosphere microbiome shifts toward combating melanose pathogen. Microbiome 10:56.

7. Andrews JM. 2001. Determination of minimum inhibitory concentrations. Journal of Antimicrobial Chemotherapy 48:5–16.

8. Mitra S, Pramanik K, Sarkar A, Ghosh PK, Soren T, Maiti TK. 2018. Bioaccumulation of cadmium by Enterobacter sp. and enhancement of rice seedling growth under cadmium stress. Ecotoxicology and Environmental Safety 156:183–196.

9. Feng J, Shentu J, Zhu Y, Tang C, He Y, Xu J. 2020. Crop-dependent root-microbe-soil interactions induce contrasting natural attenuation of organochlorine lindane in soils. Environ Pollut 257:113580.

10. Fan K, Delgado-Baquerizo M, Zhu Y, Chu H. 2020. Crop production correlates with soil multitrophic communities at the large spatial scale. Soil Biology and Biochemistry 151:108047.

11. Benjamini Y, Hochberg Y. 1995. Controlling the False Discovery Rate: A Practical and Powerful Approach to Multiple Testing. Journal of the Royal Statistical Society: Series B (Methodological) 57:289–300.

12. Fan K, Delgado-Baquerizo M, Guo X, Wang D, Zhu Y, Chu H. 2021. Biodiversity of key-stone phylotypes determines crop production in a 4-decade fertilization experiment. ISME J 15:550–561.

13. Csardi G, Nepusz T. 2006. The igraph software package for complex network research. InterJournal, complex systems 1695:1–9.

14. Bastian M, Heymann S, Jacomy M. 2009. Gephi: An Open Source Software for Exploring and Manipulating Networks. ICWSM 3:361–362.
